# Editorial: Recent advances in diagnosis, prognosis, and therapy of oncogenic virus-driven tumors

**DOI:** 10.3389/fonc.2024.1402877

**Published:** 2024-04-10

**Authors:** Mauro Tognon, Fernanda Martini, John Charles Rotondo, Giuseppe Fiume

**Affiliations:** ^1^ Department of Medical Sciences, University of Ferrara, Ferrara, Italy; ^2^ Department of Experimental and Clinical Medicine, University of Catanzaro “Magna Graecia”, Catanzaro, Italy

**Keywords:** tumor, oncogenic viruses, tumor suppressor gene, activated oncogene, diagnosis, prognosis, therapy

In recent years, the understanding of the molecular mechanisms underlying virus-induced tumorigenesis has grown exponentially, leading to breakthroughs in diagnosis, prognosis, and therapy. The development of cancer malignancy is multifaceted, involving a complex interplay of various factors rather than relying solely on one aspect. This intricate process encompasses genetic mutations, epigenetic modifications, environmental factors, and lifestyle choices. While numerous factors play a role in cancer, it is noteworthy that certain viruses, known as oncogenic viruses, can drive the cancer onset and progression in humans (1, 2). Remarkably, oncogenic viruses are implicated in a notable proportion of human cancers, accounting for >20% of cases across different cancer types (3, 4).

Oncogenic viruses are commonly classified as either DNA or RNA viruses. Notable viral agents, including high-risk types of human papillomavirus (HPV) (5), human polyomaviruses (HPyV) (6), Epstein-Barr virus (EBV), hepatitis B virus (HBV), hepatitis C virus (HCV), human immunodeficiency virus type 1 (HIV-1), human T cell lymphotropic virus type 1 (HTLV-1), and human herpesvirus type 8 (HHV-8 or Kaposi’s sarcoma herpesvirus, KSHV), have been designated as human carcinogens (Group 1) by the International Agency for Research on Cancer (IARC) (https://monographs.iarc.who.int/list-of-classifications/), while additional viruses, such as Merkel cell polyomavirus (MCPyV), are currently considered as probably carcinogenic in humans (Group 2A). Furthermore, several other viruses have been postulated, to varying degrees, to potentially contribute to oncogenesis. Of note, patients affected by immune system impairments, such as those immune-suppressed/immune-compromised, are more prone to develop oncogenic virus-driven tumors because unable to counteract in full, with their immune system, the oncogenic potential of DNA and RNA tumor viruses.

Viruses are recognized for their ability to replicate by hijacking host mechanisms to serve their own purposes. Oncogenic viruses possess the unique ability to induce tumorigenesis, facilitating the spread of malignancy from infected cells to adjacent healthy cells (1, 3, 4). The process by which oncogenic viruses contribute to the cancer onset involves the orchestration of various cofactors (7). These include the sustained presence of inflammation, the suppression of the immune system that target cancer cells, and the activation of mutagens known to promote the cancer development. Inflammatory signals from infected cells serve as a conduit for viral tumorigenesis. Upon detection, the immune system’s white blood cells are mobilized to eliminate the infected cells, thus initiating the process of clearance and repair. However, in cases of persistent or chronic inflammation, this regulatory mechanism becomes dysregulated, resulting in unabated accumulation of DNA damage. This unchecked accumulation of DNA damage predisposes cells to undergo multiple mutational events, creating a fertile environment conducive to the initiation and progression of cancer (8, 9). Oncogenic viruses target a multitude of host-signaling pathways that govern cell growth and expansion. These pathways include the phosphatidylinositol-3-kinase (PI3K)–AKT (protein kinase B)–mammalian target of rapamycin (mTOR), p53, MAPK, and WNT/β-catenin signaling pathways (4), in addition to NF-κB (10–12) and Notch signaling (13).

These molecular events regulate host cell proliferation and apoptosis, thereby facilitating cell transformation. Enhanced comprehension of the molecular mechanisms, pathways, and key players underlying the oncogenic activity of these viruses holds the potential to deepen our understanding of the oncogenic process. New knowledges on the mechanism of action of oncogenic virus-driven tumors allowed to identify specific biomarkers for an early diagnosis and targets for innovative therapies, such as new monoclonal antibodies, new class of drugs employed in pharmacotherapies, new vaccines. Altogether these new tools were employed in the precision/personalized medicine, improving oncological patient’s treatments and outcome. Investigations carried out on oncogenic virus-driven tumors gave significant results in the field of oncology and virology, allowing to set up therapeutic approaches addressed to specific targets.

This Research Topic comprises five articles, including two original research articles, one meta-analysis report, one case report and one review, focusing on different aspects of viral oncogenesis. Specifically, Zheng et al. explore the role of T antigen of JC polyomavirus (JCPyV), which belongs to the *polyomaviridae* family (6) and is considered as an etiologic agent of progressive multifocal leukoencephalopathy (PML). In their study, Zheng et al. report that the T antigen knockdown resulted in the suppression of proliferation, glycolysis, mitochondrial respiration, migration, and invasion, while inducing apoptosis and G2 arrest. Conversely, the T antigen overexpression led to increased expression of Akt, survivin, retinoblastoma protein, β-catenin, β-transducin repeat-containing protein (TRCP), and inhibitor of growth (ING)1, and led to the downregulation of both total content and phosphorylated form of mTOR, p-p38, Cyclin D1, p21, vascular endothelial growth factor (VEGF), ING2, and ING4 in both hepatocellular and pancreatic cancer cells and tissues. Furthermore, the T antigen knockdown elicited a comprehensive alteration in transcriptome, metabolome, and proteome. These findings significantly contribute to the elucidation of multiple host pathways impacted by JCPyV T antigen.

In their work, Klufah et al. conducted an analysis of decoy cells present in urine specimens obtained from immunocompetent individuals, with and without diagnosed urothelial cell carcinomas (UCC). Among the 30 patients exhibiting decoy cells, 14 had previously received a diagnosis of UCC of the urinary bladder (14/30; 46.6%) prior to the detection of decoy cells in their urine samples. Klufah et al. report the presence of JCPyV in the urine specimens and UCC cases of immunocompetent patients. Additionally, Merkel cell polyomavirus (MCPyV) was identified in two UCC cases. Notably, a total of five UCC cases exhibited the presence of either JCPyV or MCPyV. These findings support to the hypothesis that these viruses with oncogenic potential may occasionally be associated with UCC occurrence. However, further comprehensive studies are warranted to validate the significance of JCPyV or MCPyV as potential risk factors contributing to the UCC development.

Liao et al. report a compelling case involving a 70-year-old male patient diagnosed with stage IV advanced intrahepatic cholangiocarcinoma (ICC). Notably, the patient exhibited a remarkably high tumor mutational load and hyper-expression of programmed death-ligand 1 (PD-L1) gene (90%), alongside positive Epstein-Barr virus-encoded RNA (EBER). The treatment regimen employed the PD-1 inhibitor Pembrolizumab in conjunction with radiotherapy. Remarkably, this approach resulted in significant shrinkage and inactivation of the primary tumor foci, coupled with the disappearance of metastases, ultimately culminating in complete remission for the patient. These findings underscore the potential of combining PD-1 inhibitors with radiotherapy as a promising therapeutic strategy for managing this specific cancer.

In their comprehensive meta-analysis, Chen et al. scrutinized various studies involving 886 patients affected by lung cancer. Their findings revealed a noteworthy association between EBV infection and a more than four-fold increase in the risk of developing lung cancer. Importantly, this heightened risk was found to be correlated with specific pathological characteristics, including lymphatic infiltration and degree of differentiation, particularly within the rare subtype of pulmonary lymphoepithelioma in non-small cell lung cancer (NSCLC). Furthermore, the analysis unveiled racial and regional disparities in the prevalence of EBV-infected lung cancer, with the Asian population exhibiting a higher susceptibility to this correlation.

In their comprehensive review, He and Tang explore a diverse range of research findings concerning preoperative and postoperative predictors of hepatectomy for HBV-related hepatocellular carcinoma (HCC) spanning the past decade, as well as insights gleaned from landmark studies predating this timeframe. The review delves into pertinent aspects of HCV-related HCC, non-HBV non-HCV HCC, and the burgeoning application of artificial intelligence within this field.

In their study, Vecchio et al. explored the potential of IBTK silencing as a therapeutic approach for aggressive B-lymphomas, especially when combined with the chemotherapeutic agent Rituximab. Rituximab, in combination with chemotherapy (R-CHOP), serves as the standard first-line therapy for non-Hodgkin’s lymphoma (NHL). Vecchio et al. investigated the impact of IBTK on the expression of the oncogene MYC in B-lymphoma. Employing cell culture models, they evaluated the effects of Rituximab combined with IBTK silencing on cell viability through cell cycle analysis and Annexin V assays. Furthermore, they explored the therapeutic potential of IBTK inhibition in vivo using a mouse model of Eμ-myc lymphomas. Their results indicate that IBTK silencing enhances the anti-tumor effects of Rituximab, leading to apoptosis in aggressive B-lymphomas both in vitro and in vivo. This study highlights the promising role of IBTK as a target for novel therapeutic strategies in the treatment of aggressive B-lymphomas, particularly in combination with Rituximab.

In conclusion, the collective findings presented in this Research Topic offer a deep insight into the multifaceted landscape of virus-induced tumorigenesis. Through a meticulous examination of molecular mechanisms, host-pathogen interactions, and therapeutic interventions, these studies have not only expanded our understanding of viral oncogenesis, but also provided a foundation for innovative diagnostic and therapeutic strategies. From elucidating the role of viral antigens and host pathways in cancer initiation and progression to exploring the potential of immunotherapy and radiotherapy in combating virus-driven tumors, each contribution represents a significant step forward in the fight against cancer. Moving forward, continued interdisciplinary research and collaboration will be essential to translate these discoveries into tangible benefits for patients, ultimately paving the way towards more effective prevention, diagnosis, and treatment of virus-associated cancers.

## Author contributions

MT: Writing – original draft, Writing – review & editing. FM: Writing – original draft, Writing – review & editing. JCR: Writing – original draft, Writing – review & editing. GF: Writing – original draft, Writing – review & editing.
